# The Effect of Pregabalin-Paracetamol on Shoulder Pain in Patients Following Laparoscopic Cholecystectomy: A Randomized Clinical Trial

**DOI:** 10.5812/aapm-149328

**Published:** 2025-11-15

**Authors:** Javad Shahinfar, Hossein Zeraati, Ali Esmaeili, Yasmin Ghelichi, Mahsa Hossein Zadeh, Mohsen Rakhsha, Hosseinali Soltani

**Affiliations:** 1 Department of Anesthesiology, Emam Ali Hospital, North Khorasan University of Medical Sciences, Bojnurd, Iran; 2 Department of Surgery, Emam Ali Hospital, North Khorasan University of Medical Sciences, Bojnurd, Iran

**Keywords:** Cholecystectomy, Shoulder Pain, Pregabalin, Paracetamol, Laparoscopy

## Abstract

**Background:**

Shoulder pain is widely recognized as one of the most prevalent complications following cholecystectomy procedures. The management of postoperative shoulder discomfort primarily relies on pharmacological interventions. Pregabalin, a commonly prescribed medication, is valued for its efficacy in modulating neuropathic pain.

**Objectives:**

This study aimed to evaluate the impact of preoperative pregabalin, in combination with paracetamol, on the control of shoulder pain after cholecystectomy.

**Methods:**

This randomized, double-blind clinical trial enrolled 90 patients aged 20 - 60 years, scheduled for cholecystectomy at Imam Ali Hospital in Bojnourd in 2023. Patients were selected by convenience sampling and randomly assigned to one of three groups. The first group received 300 mg of oral pregabalin one hour before surgery, as well as 1 g of intravenous paracetamol 30 minutes before the end of the operation, followed by dosing every 6 hours for 24 hours. The second group received only oral pregabalin as premedication one hour prior to surgery. The third group received standard care (standard multimodal analgesia with diclofenac as needed), without pregabalin or paracetamol. The primary outcome was the severity of shoulder pain during recovery and at 6, 12, 18, and 24 hours postoperatively, measured using the Visual Analog Scale (VAS).

**Results:**

Significant differences were observed in the severity of postoperative shoulder pain among the three groups at all-time intervals (P < 0.05). Inter-group comparisons revealed that the severity of shoulder pain in the first group was significantly lower than in the third group at all-time points (P < 0.05). Additionally, the second group exhibited significantly lower shoulder pain severity compared to the third group at all-time points except at 18 and 24 hours postoperatively (P < 0.05). There was a notable reduction in pain severity over time in the first and second groups (P < 0.001).

**Conclusions:**

According to the study findings, premedication with oral pregabalin and intravenous paracetamol effectively alleviated postoperative shoulder pain across all time intervals without adverse effects. Pregabalin premedication alone also demonstrated analgesic effects, though with a shorter duration compared to the combination regimen.

## 1. Background

Laparoscopy is a minimally invasive and widely used technique for diagnosing and treating a variety of clinical conditions. Although it is considered an efficient and preferred surgical approach, it has certain drawbacks, with postoperative shoulder pain being one of the most common. Residual carbon dioxide and peritoneal or diaphragmatic irritation stimulate the phrenic nerve (C3 - C5), producing referred shoulder pain; the severity is related to insufflation pressure and residual gas volume (1). Several studies have reported the incidence of this complication to range from 60% to 80%. This pain may result in prolonged discomfort for patients, extended hospital stays, and increased medical expenses (1, 2).

Various strategies have been examined for the management of post-laparoscopic shoulder pain, with pharmacological interventions emerging as some of the most effective approaches (3-5). Pregabalin, a member of the gabapentin analog class, has demonstrated efficacy in the treatment of fibromyalgia and neuropathic pain. Several studies have underscored its beneficial effects in managing neuropathic pain, and pregabalin has shown promise in alleviating localized pain associated with laparoscopic surgeries. Research has provided evidence of its ability to effectively control severe and chronic pain, consequently reducing opioid requirements for up to 24 hours following surgery (6, 7).

Paracetamol (acetaminophen), a non-opioid analgesic and antipyretic, exhibits anti-inflammatory, analgesic, and antipyretic properties. Widely used in adult pain management, paracetamol has proven effective in reducing the need for opioids in postoperative settings. In addition, studies have demonstrated its positive impact and efficacy in the treatment of chronic non-cancer pain (8, 9).

Despite the advantages of laparoscopy, complications such as shoulder pain may lead to extended hospitalizations. Previous studies have examined pharmacological interventions involving pregabalin and paracetamol separately for various pain conditions, with positive outcomes. However, the combined effect of these two drugs on pain management remains unexplored.

## 2. Objectives

This study aims to investigate the comparative effectiveness of their simultaneous administration in alleviating post-laparoscopic shoulder pain.

## 3. Methods

### 3.1. Study Population

This randomized, double-blind clinical trial was conducted with 90 patients aged 20 - 60 years who were candidates for laparoscopic cholecystectomy at Imam Ali (AS) Teaching Hospital in Bojnourd in 2023. Inclusion criteria were: Age 20 - 60 years, physical status grades I or II according to the American Society of Anesthesiologists (ASA), no history of drug addiction, chronic pain, liver, psychiatric, or kidney disease, no allergy to pregabalin or paracetamol, and no history of shoulder or chest surgery. Exclusion criteria included conversion to open surgery and any clinical condition or complication that could worsen postoperative pain.

### 3.2. Sample Size and Randomization Method

Sample size was calculated as


$$n\;=\;2{\left(Z_{\frac{\alpha }{2}}+Z_{\beta }\right)}^{2}\times \frac{\sigma^{2}}{{∆}^{2}}$$

(two‑sided α = 0.05, power = 80%). Assuming σ = 2.0 and Δ = 1.5 Visual Analog Scale (VAS) units from prior laparoscopic cholecystectomy trials, n = 27 per group; this was increased to 30 per group to account for attrition. Convenience sampling was used, and participants were divided into three groups via blocked randomization using a web-based system (https://www.randomizer.org/), which created 15 blocks of six participants each. Within each block, two participants were allocated to groups A, B, and C. Group assignments were labeled on cards (A, B, or C), sequentially numbered from 1 to 90 by a person not involved in the research, and placed in sealed envelopes. The envelopes were shuffled and opened only upon each patient’s arrival to ensure allocation concealment.

### 3.3. Anesthesia Induction and Maintenance

On entering the operating room, ASA standard monitoring was applied (ECG, non-invasive blood pressure, SpO_2_, and capnography); end-tidal CO_2_ was maintained at 35 - 45 mmHg with controlled ventilation. Anesthesia induction and maintenance were standardized across groups. Pre-anesthetic medication included midazolam (2 mg) and fentanyl (1 - 2 μg/kg). Anesthesia was induced with propofol (1.5 - 2.5 mg/kg), and neuromuscular blockade was achieved using atracurium (0.4 - 0.6 mg/kg). Maintenance anesthesia was achieved with isoflurane inhalation (1.2 - 1.5 vol%). Intraoperative analgesia included induction fentanyl (1 - 2 μg/kg); titrated boluses (0.5 - 1 μg/kg) were permitted as needed and totals were recorded. Reduced-dose atracurium maintained muscle relaxation, with reversal using neostigmine and atropine.

### 3.4. Surgical Procedure

Laparoscopic port placement and insufflation with carbon dioxide were tailored to patient needs. Intra-abdominal pressure was initially set at 8 - 10 mmHg, then increased to 12 - 15 mmHg as required. Laparoscopic cholecystectomy was performed with patients positioned in reverse Trendelenburg with a left lateral tilt. At the end of the procedure, carbon dioxide was evacuated and trocar sites were closed.

### 3.5. Intervention

Participants were randomly assigned to three groups:

- Group 1: Received oral pregabalin (300 mg) one hour preoperatively, and intravenous paracetamol (1 g/100 mL normal saline) 30 minutes before the end of surgery, followed by 1 g/100 mL every 6 hours for 24 hours (maximum total 4 g/day).

- Group 2: Received oral pregabalin (300 mg) one hour preoperatively and intravenous normal saline (placebo) 30 minutes before the end of surgery, followed by normal saline infusions every 6 hours postoperatively.

- Group 3: Received routine care, including oral placebo preoperatively and intravenous normal saline on the same schedule as group 2 for blinding purposes.

### 3.6. Outcome Assessment

In the recovery room, sedation and comfort were assessed using the Ramsay Sedation Scale. For shivering, 25 mg intravenous midazolam was administered. A uniform postoperative pain management protocol was applied in all groups: Diclofenac suppositories (100 mg) were administered as needed for pain control during hospitalization. Rescue protocol: Diclofenac was administered for VAS ≥ 5 or on request; if pain persisted or VAS ≥ 7, intravenous morphine (2 - 3 mg boluses) was given. During the first 24 hours post-surgery, side effects such as itching, nausea, vomiting, urinary retention, and dizziness were recorded. The severity of nausea and vomiting was measured by VAS. Ondansetron (8 mg IV) was administered for nausea or vomiting, and the number of patients requiring this medication was noted. Morphine use for pain scores greater than 7 was recorded. Shoulder pain was assessed using the VAS at recovery and at 6, 12, 18, and 24 hours postoperatively.

### 3.7. Blinding

To ensure allocation concealment, sealed opaque envelopes were opened upon each patient’s arrival. In groups 2 and 3, saline infusions replaced paracetamol for blinding. Patients were unaware of their group allocation. Drug preparation for injection was performed by a non-participant, and administration was by the relevant nurse. All research team members and outcome assessors remained blinded to group assignments.

### 3.8. Data Analysis

Data normality was assessed using the Shapiro-Wilk test. Quantitative data were presented as mean ± standard deviation (SD) and analyzed by one-way analysis of variance (ANOVA), with post-hoc tests for inter-group comparisons as needed. Univariate ANOVA was used to control potential confounders. Categorical variables were expressed as frequency and percentage, and compared using the chi-square test or Fisher’s exact test as appropriate.

### 3.9. Ethical Considerations

This study was approved by the Ethics Committee of North Khorasan University of Medical Sciences (IR.NKUMS.REC.1400.019). It was registered as a double-blind clinical trial in the Iranian Registry of Clinical Trials (IRCT20141001019359N11), with peer review board approval. Study objectives were explained in simple language to participants, and written informed consent was obtained from all when fully alert. Participation was voluntary, and patients could withdraw at any time.

## 4. Results

**Figure 1.**
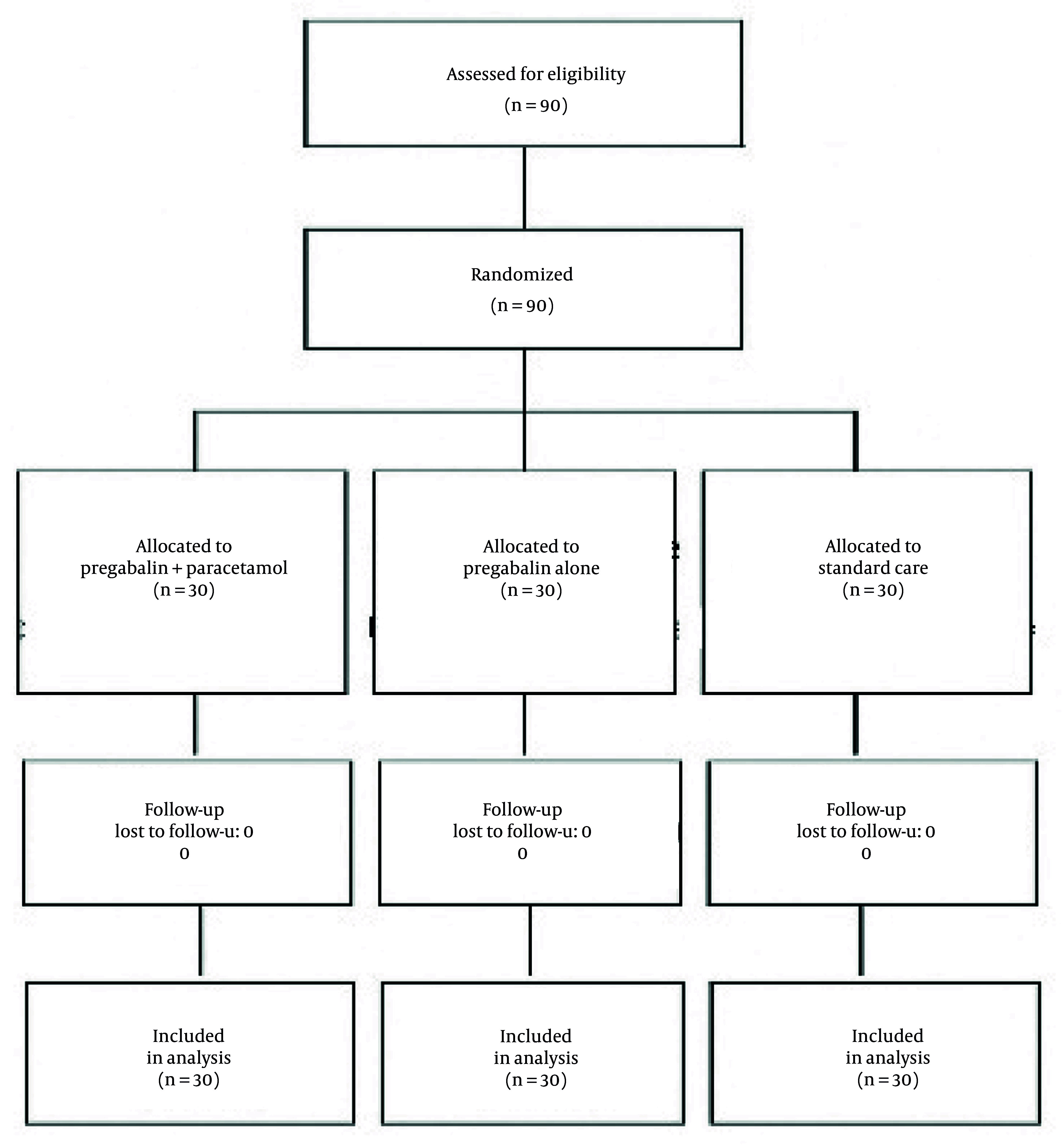
Flow chart of CONSORT

Ninety participants were enrolled and distributed equally among three groups (Figure 1). 

Table 1 presents the demographic data of the participants. No significant differences were found among the groups in gender (P = 0.761), age (P = 0.48), weight (P = 0.31), fasting duration (P = 0.76), or surgery duration (P = 0.21). Thus, the groups were homogeneous across these parameters.

**Table 1.** Comparison of Demographic Parameters in the Three Study Groups ^a^

Parameters	Paracetamol Pregabalin	Pregabalin	Control	P-Value
**Gender**				
Man	17 (6.56)	16 (3.53)	15 (50)	0.761 ^b^
Female	13 (4.43)	14 (7.46)	15 (50)	
**Age (y)**	51.2 ± 4.45	49.1 ± 4.95	52.6 ± 3.8	0.48 ^c^
**Weight**	70.5 ± 5.2	73.8 ± 5.7	75.8 ± 6.4	0.21 ^c^
**Duration of fasting**	9.5 ± 0.8	10.1 ± 1.4	9.9 ± 1.0	0.76 ^c^
**Duration of surgery**	52.4 ± 10.3	45.7 ± 8.7	48.4 ± 9.5	0.21 ^c^

^a^ Values are expressed as No. (%) or mean ± SD.

^b^ Chi-square test.

^c^ One-way analysis of variance (ANOVA) test.

The one-way ANOVA results in Table 2 demonstrated significant differences in postoperative shoulder pain severity across all groups and at all-time intervals (P < 0.05). Intra-group comparisons showed a significant reduction in shoulder pain scores over time in the first and second groups (P < 0.001), while the third group showed no significant change (P = 0.5).

**Table 2.** Comparison of the Average Shoulder Pain Score in the Three Study Groups at Different Time Periods According to the Visual Analog Scale ^a,^ ^b^

Time Frames	Paracetamol+Pregabalin	Pregabalin	Control Group	P-Value
**Recovery**	2.1 ± 5.3	4.1 ± 5.4	0.2 ± 0.6	0.021
**6 h**	0.8 ± 1.3	0.9 ± 2.4	7.1 ± 5.5	0.017
**12 h**	0.6 ± 3.2	0.1 ± 8.3	4.1 ± 9.4	0.034
**18 h**	0.5 ± 1.2	0.8 ± 5.3	4.1 ± 0.4	0.039
**24 h**	0.4 ± 7.1	0.6 ± 9.2	1.1 ± 7.3	0.041

^a^ Values are expressed as mean ± SD.

^b^ One-way analysis of variance (ANOVA) test.

**Figure 2.**
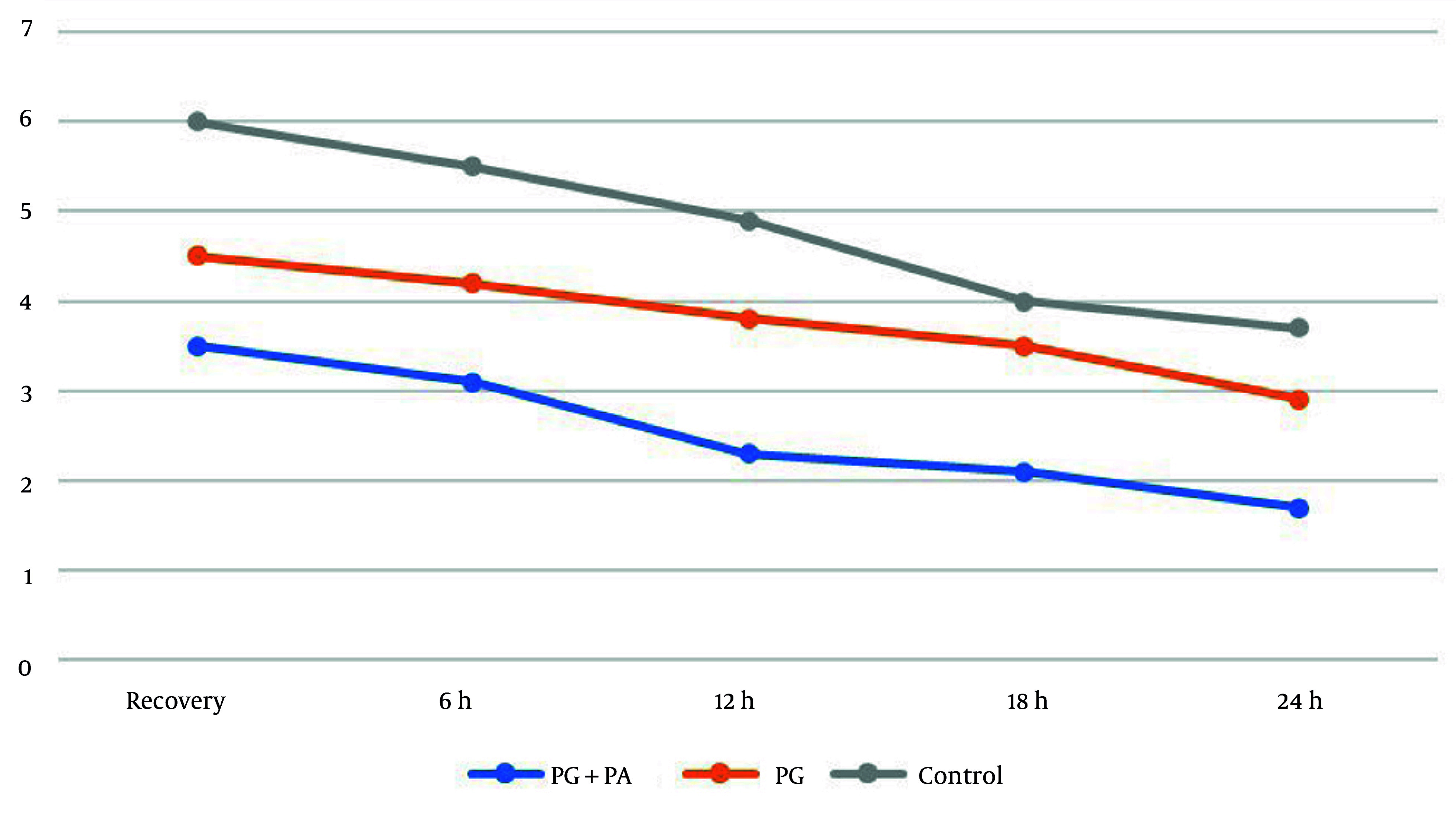
Comparison of the average shoulder pain score in the three study groups at different time periods according to the Visual Analog Scale (VAS)

Given the significant disparities in the one-way ANOVA, post-hoc tests were performed for pairwise comparisons. The findings indicated no significant difference between the second and third groups at 18 hours (P = 0.063) and 24 hours (P = 0.052). In all other time points, shoulder pain scores were significantly lower in the first group compared to the second group, and significantly lower in the second group compared to the third group (Figure 2). 

**Figure 3.**
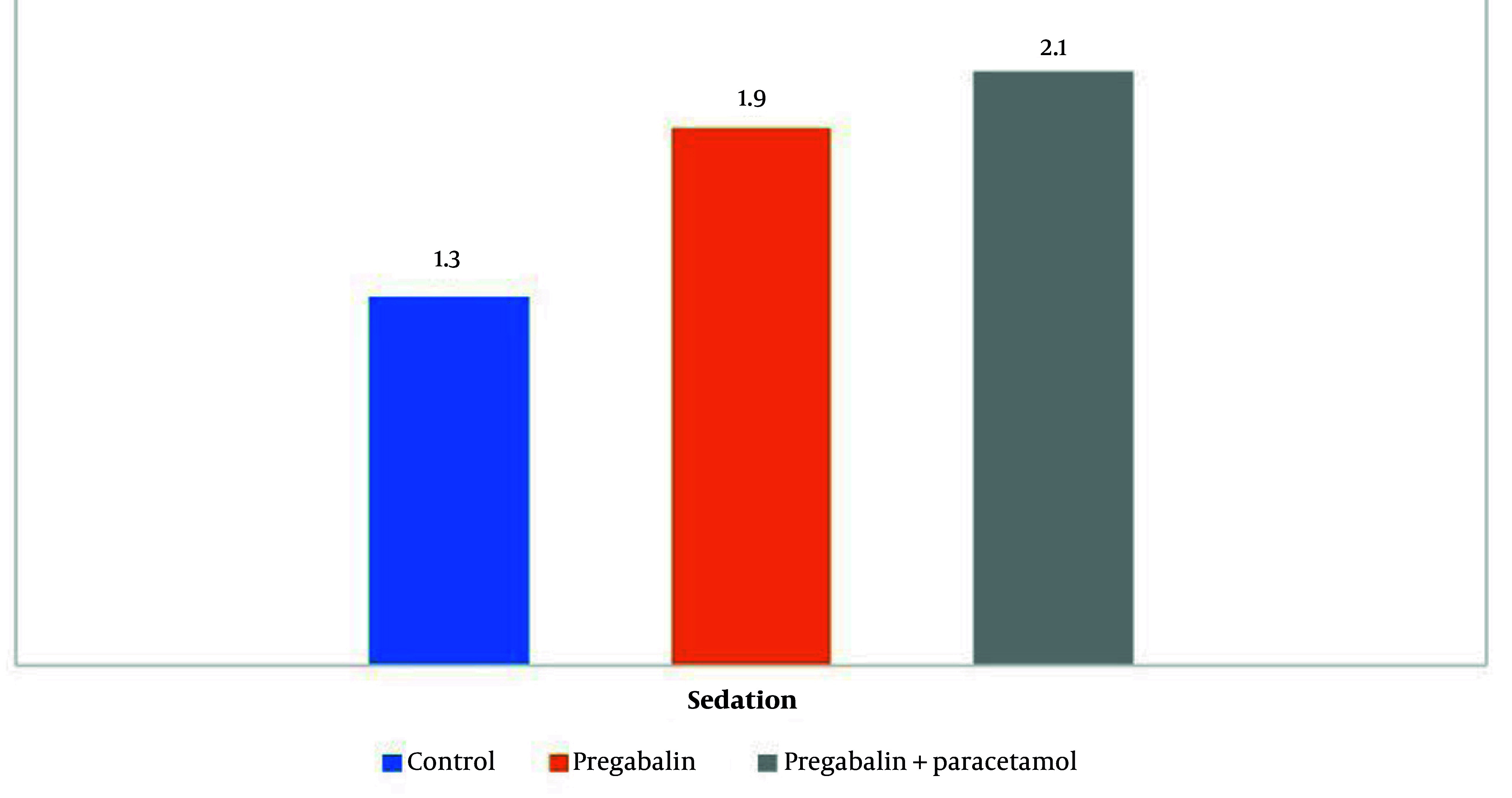
Comparison of the average sedation score in the three study groups

The mean sedation score in the first and second groups was significantly higher than in the third group (P = 0.017), with no statistically significant difference between the first and second groups (P = 0.06, Figure 3). 

The mean dose of morphine consumed in the first group (2.1 ± 5.3 mg) was significantly lower than in the second (6.2 ± 4.6 mg) and third groups (6.4 ± 8.9 mg, P < 0.001). Comparison of clinical adverse events among the three groups is shown in Table 3. No significant differences in clinical adverse events were observed among the groups.

**Table 3.** Comparison of the Frequency of Postoperative Clinical Complications in the Three Study Groups

Variables	Paracetamol+Pregabalin	Pregabalin	Control	P-Value
**Dizziness**	2	2	1	0.08
**Urinary retention**	2	1	2	0.74
**Itching**	0	1	1	0.89
**Shivering**	3	5	4	0.46
**Severe nausea**	1.4 ± 3.3	1.3 ± 3.7	1.8 ± 3.9	0.23
**Vomit**	0	0	1	0.76

## 5. Discussion

Shoulder pain is a common complication following laparoscopic surgeries and can significantly impact patients’ quality of life. Therefore, implementing pain management strategies, particularly pharmacological interventions, is essential for reducing postoperative pain (1, 3, 4, 10). This study was designed to evaluate the efficacy of 300 mg of oral pregabalin in conjunction with intravenous paracetamol infusion for managing postoperative shoulder pain after laparoscopic cholecystectomy.

The results demonstrated that the administration of 300 mg oral pregabalin with paracetamol infusion substantially decreased postoperative shoulder pain. Pregabalin is primarily used to treat seizures and convulsions but is also utilized for its analgesic and anxiolytic properties in perioperative settings. Pregabalin’s pharmacokinetic profile is favorable, with a half-life of 6 - 8 hours, bioavailability of 90%, and peak plasma concentrations achieved within 30 - 120 minutes (11-13).

Multiple studies have established the efficacy of gabapentinoids as adjuncts in postoperative pain reduction, particularly following laparoscopic, hysterectomy, and mastectomy procedures (14, 15). Preoperative pregabalin doses typically range from 150 to 300 mg, with no reported adverse effects postoperatively (15). Systematic reviews and meta-analyses in patients undergoing laparoscopic surgeries suggest that pregabalin’s analgesic effect persists for 4 - 5 days postoperatively, compared to other gabapentinoids (16). Eidy et al. reported lower levels of postoperative pain and reduced pethidine requirement in patients treated with pregabalin, supporting its use as premedication (17). Some challenges with pregabalin include the potential for postoperative side effects such as dizziness and blurred vision, particularly at higher doses (15, 18); however, this study observed no significant drug-related adverse effects with a 300 mg dose.

Supporting these findings, Nakhli et al. reported a significant decrease in shoulder pain intensity for up to 48 hours postoperatively with either 150 mg of pregabalin or 600 mg of gabapentin (19). Additional benefits included reduced nausea and vomiting, faster unassisted ambulation, and improved sleep quality, with no notable differences between the two dosages (19, 20). Mishra et al. confirmed a marked reduction in pain and decreased need for additional analgesics with 150 mg pregabalin after laparoscopic surgery (13). Bekawi et al. found that 150 mg pregabalin preoperatively significantly reduced pain scores and pethidine requirements (20). Sarakatsianou et al. showed that 600 mg pregabalin premedication significantly reduced pain at rest and with movement, as well as morphine requirements (18). Valadan et al. demonstrated that preoperative gabapentin (600 mg) significantly reduced shoulder pain intensity at rest and with movement for up to 12 hours postoperatively in patients undergoing laparoscopic ovarian surgery (21).

Nevertheless, the analgesic effects of pregabalin have been inconsistent across studies (11, 22). Some research has shown that pregabalin premedication for shoulder pain within the first 6 hours postoperatively did not differ significantly from controls (11). A systematic review concluded that pregabalin’s main analgesic effect begins from the second postoperative hour (22). In the present study, single-dose pregabalin provided superior analgesia for up to 12 hours postoperatively, with no significant effect beyond that period, likely due to its short half-life (4.6 - 6.8 hours) following single administration.

A limitation of pregabalin is postoperative side effects (7, 23, 24). One study indicated an increase in dizziness following pregabalin premedication after laparoscopic cholecystectomy. In this study, intravenous paracetamol (1 g) combined with oral pregabalin (300 mg) significantly reduced shoulder pain intensity for up to 24 hours postoperatively (18).

Upadya et al. corroborated these findings, demonstrating that intravenous paracetamol extended shoulder pain control compared to intramuscular 0.5% bupivacaine in patients undergoing laparoscopic cholecystectomy (25). Esmat and Farag showed that 1 g oral paracetamol significantly reduced pain compared to 150 and 300 mg oral pregabalin within the first 30 minutes to 6 hours postoperatively, although after 6 hours, analgesic effects in all groups were comparable (26). Choudhuri and Uppal demonstrated that intravenous paracetamol (1 g) significantly reduced postoperative pain intensity in patients also receiving intravenous fentanyl (27). Collectively, these results affirm the effectiveness of paracetamol for postoperative shoulder pain, with the added advantage of a lack of reported adverse effects.

This study found that oral pregabalin premedication increased sedation scores, whereas paracetamol had no effect on sedation, consistent with previous research (13, 20, 27, 28). However, Esmat and Farag reported increased sedation with paracetamol and pregabalin in the first 2 hours post-induction (26). Variations may be attributable to differences in drug dosages and timing of parameter measurement. Singh et al. showed that 150 mg pregabalin produced greater sedation than 300 mg, while Asgari et al. found that 300 mg pregabalin was more effective than 50 and 75 mg doses (15, 29).

Pregabalin premedication was associated with significantly reduced postoperative morphine requirements. The addition of intravenous paracetamol further diminished postoperative pain, consistent with earlier studies. Singh et al. reported a significant decrease in morphine requirements with 150 mg pregabalin compared to 300 mg in the first 8 hours postoperatively, but no difference between 150 mg and 300 mg in the 8 - 24 hour period (15). Balaban et al. demonstrated that 300 mg pregabalin was more effective in reducing fentanyl consumption in the first 30 minutes after surgery than 150 mg, but both doses were similarly effective thereafter (30). Agrawal et al. found that 300 mg pregabalin significantly reduced postoperative fentanyl use (31). Paracetamol has also been shown to extend the time to first analgesic requirement or reduce total dose needed.

No significant differences in adverse effects were observed among the groups, consistent with previous research on nausea, vomiting, itching, urinary retention, and dizziness. However, Esmat and Farag (26) found a higher incidence of vomiting in patients using both paracetamol and pregabalin compared to pregabalin alone, contrary to the present findings.

The double-blind design and standardized anesthesia support internal validity of this study; however, the single-center setting, ASA I-II selection, and 24-hour follow-up limit generalizability. Future research should evaluate lower pregabalin doses in combination regimens and adopt uniform rescue thresholds (VAS ≥ 4) with longer follow-up.

The present results demonstrate that preoperative administration of pregabalin with paracetamol in patients undergoing laparoscopic cholecystectomy significantly reduces shoulder pain severity within 24 hours postoperatively. Therefore, it is recommended to prescribe oral pregabalin premedication alongside intravenous paracetamol infusion for extended postoperative pain control.

## Data Availability

The dataset presented in this study is available on request from the corresponding author during submission or after publication. The data are not publicly available due to privacy or ethical restrictions.

## Clinical Trial Registration Code:

IRCT20141001019359N11.
